# Welcome to a New Volume of Advanced Science

**DOI:** 10.1002/advs.202207095

**Published:** 2023-01-04

Following the trend of the past few years, the year 2022 was largely dominated by COVID restrictions. While not many personal meetings took place between the editorial team and our authors and readers, we found other ways to interact: as promised in our editorial of the previous year, in March 2022, *Advanced Science* and its sister journal, *Small Science*, hosted two virtual events. These “**Spotlights in *Advanced Science* and *Small Science*”** came in two parts. In the first part, Zhenan Bao (Stanford University, USA), Ali Khademhosseini (Terasaki Institute, Los Angeles, USA), and Hua Kuang (Jiangnan University, Wuxi, China) focused on Advances in Biomedical Research; in the second part, Shengzhong Liu (Chinese Academy of Sciences, Dalian, China), Arumugam Manthiram (University of Texas at Austin, USA), and Stefan Hecht (DWI Leibniz Institute for Interactive Materials, Aachen, Germany) covered energy research and smart materials. The webinars were extremely well attended, and the feedback was entirely postive. Due to the different time zones of our attendees (and speakers), not everyone could join all live sessions. We therefore have a recording available via the *Advanced Science* homepage, which continues to be very popular.

**Figure 1:**
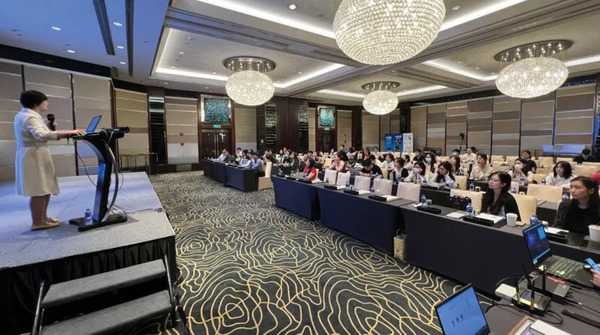
Live attendance at the *Advanced Science* Biomedical Symposium in Shanghai.

In addition to these virtual highlights, our colleagues Dr. Peijun Tu and Dr. Xi Wen organized the first ***Advanced Science* Biomedical Symposium**. The event was held both online and on‐site (in Shanghai; see **Figure** **1**) on Sep. 23^rd^, 2022. It was co‐organized by the State Key Laboratory of Genetic Engineering in Fudan University, and the on‐site symposium was supported by BioGenous Technologies. 12 top researchers (four of them board members of *Advanced Science*) gave interesting talks in the fields of metabolism, cancer, epigenetics, immunology, and chemical biology – fields that are strongly growing in *Advanced Science*. The event attracted 70 people on‐site as well as 1.9k online views and was a big success. We are looking forward to similar symposia in the future – hopefully then without COVID restrictions.

Another highlight in the middle of last year was the publication of the very first special issue in *Advanced Science*: on the occasion of Professor Klaus Müllen's 75^th^ anniversary, Tanja Weil and Andreas Herrmann (both board members of *Advanced Science*) acted as guest editors for this top collection of articles. The issue aimed to highlight Professor Müllen's achievements and impact on a wide range of areas involving functional carbon‐rich materials. With a compilation of articles from his close collaborators and former alumni, we honor a remarkable chemist, a visionary researcher, and an inspiring teacher for several generations of chemists and materials scientists worldwide, who has stimulated and shaped this research field over several decades. Check out the full issue here.

As every year, we would like to present this year's most influential papers. Excitingly, our top‐cited paper of 2022 is on cancer immunology. Batteries and solar cells continue to be popular topics in *Advanced Science*, but in 2022 we were excited to see a growing variety of research areas represented in the list, with antibacterial hydrogels, triboelectric textiles, implants, carbon dots, and nanocellulose also among the hot topics. Congratulations to the authors listed in **Table** **1**.

**Table 1:** Top 10 most‐cited *Advanced Science* articles in 2022, published in 2020–2021 (data extracted on December 16, 2022)

Title	Corresponding Author(s), Affiliation	Published in	Citations in 2021 (Web of Science)	Total Citations (Web of Science)
ImmuCellAI: A Unique Method for Comprehensive T‐Cell Subsets Abundance Prediction and its Application in Cancer Immunotherapy	An‐Yuan Guo, Huazhong University of Science and Technology, Wuhan, China	February 2020	172	326
Progress and Perspective of Ceramic/Polymer Composite Solid Electrolytes for Lithium Batteries	Yan‐Bing He, Tsinghua University, Shenzhen, China	January 2020	108	246
Highly Stretchable, Adhesive, Biocompatible, and Antibacterial Hydrogel Dressings for Wound Healing	Bingna Zheng, Dingcai Wu, and Hui Wang, Sun Yat‐sen University, Guangzhou, China	March 2021	98	134
An Artificial Polyacrylonitrile Coating Layer Confining Zinc Dendrite Growth for Highly Reversible Aqueous Zinc‐Based Batteries	Lijun Fu, Yuping Wu, and Xianwei Hu, Nanjing Tech University, China and Northeastern University, Shenyang, China	March 2021	96	123
Progress in Stability of Organic Solar Cells	Leiping Duan and Ashraf Uddin, University of New South Wales, Sydney, Australia	April 2020	92	176
Machine Learning Glove Using Self‐Powered Conductive Superhydrophobic Triboelectric Textile for Gesture Recognition in VR/AR Applications	Ting Zhang and Chengkuo Lee, Chinese Academy of Sciences, Suzhou, China and National University of Singapore	June 2020	88	176
Solutions to the Drawbacks of Photothermal and Photodynamic Cancer Therapy	Zengwu Shao and Yanli Zhao, Huazhong University of Science and Technology, Wuhan, China and Nanyang Technological University, Singapore	January 2021	89	124
Biodegradable Magnesium‐Based Implants in Orthopedics—A General Review and Perspectives	Jia‐Li Wang and Ling Qin, The Chinese University of Hong Kong, China	February 2020	84	155
Rational Design of Multi‐Color‐Emissive Carbon Dots in a Single Reaction System by Hydrothermal	Siyu Lu, Zhengzhou University, China	November 2020	84	127
Nanocellulose‐MXene Biomimetic Aerogels with Orientation‐Tunable Electromagnetic Interference Shielding Performance	Chuanfang (John) Zhang and Gustav Nyström, Empa, Dübendorf, Switzerland	June 2020	81	147

**Figure 2:**
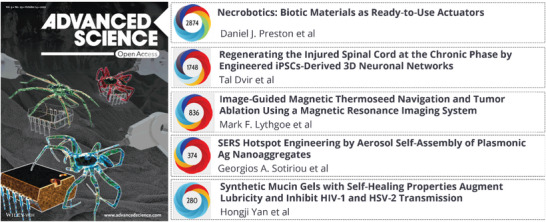
Top 5 articles listed according to their Altmetric score, published in *Advanced Science* January–November 2022.

We would also like to highlight articles published in 2022 with the highest Altmetrics scores (**Figure** **2**). The Altmetrics score is a measure of the number of times publications are mentioned by online news outlets, blogs, and social media channels. Bioinspired robotics, the regeneration of injured spinal cords, tumor imaging, plasmonic nanoaggregates for sensing applications, and gel‐based lubricants against viral transmission were the topics that generated the most publicity this year.

In last year's editorial, we announced the launch of our new open access sister journals: *Advanced Energy and Sustainability Research* , *Advanced NanoBiomed Research* and *Advanced Photonics Research* . We are very proud to let you know that these journals have developed into highly respected titles themselves and will be receiving their first impact factors already this summer. But of course we did not stop there. 2022 saw the birth of two additional ***Advanced X Research*** journals: *Advanced Physics Research* and *Advanced Sensor Research* , both of which published their first issues in December 2022. We are confident that these spin‐offs will also develop into highly attractive open‐access publication outlets for their respective communities.

Towards the end of last year, we revised the editorial advisory boards of *Advanced Science*, this time with a clear focus on top female researchers. We would like to welcome Jennifer A. Lewis (Harvard SEAS) and Hongyan Wang (Chinese Academy of Science) to our Executive Advisory Board. Sarah Heilshorn (Stanford University), Yingying Lu (Zheijiang University), Cornelia Palivan (University of Basel), Molly Shoichet (University of Toronto), Brigitte Stadtler (Aarhus University), Wenwen Zeng (Tsinghua University), and Shimei Zhuang (Sun Yat Sen University) have kindly agreed to join our International Advisory Board. We look forward to a close and fruitful collaboration.

Also internally, there have been some changes in our team. Unfortunately, our long‐serving deputy editor Dr. Prisca Henheik gave up her position to join the Food and Environmental group in Wiley. We are very grateful to Prisca for many years of hard work and great collaboration and wish her all the best in her new role. Anke Osterland (based in Weinheim, Germany), was promoted to a deputy role. For several years, Anke has already been very efficiently handling the post‐acceptance work in *Advanced Science* and is responsible for the cover design and coordination as well as for article promotion. In addition, Xi Wen (based in our Shanghai office) is our new deputy editor for bio‐ and life science areas. Four more editors joined the team over the course of last year: Dr. Flora Kiss (based in Berlin, Germany), Dr. Richard Murray (based in Spain), Dr. Mihai Peterca (based in Hoboken, USA), and Dr. Hao Wei (based in Shanghai, China). With these additional resources, we will continue to constantly improve *Advanced Science* to become your journal of choice.

We cannot end an editorial without saying thank you – to our authors, reviewers, Advisory Board members, and readers. We look forward to another exciting year full of top research and the whole team wishes you a happy, healthy, and successful year 2023!

On behalf of the whole team,

Kirsten Severing

